# Role of qSOFA and SOFA Scoring Systems for Predicting In-Hospital Risk of Deterioration in the Emergency Department

**DOI:** 10.3390/ijerph17228367

**Published:** 2020-11-12

**Authors:** Raúl López-Izquierdo, Pablo del Brio-Ibañez, Francisco Martín-Rodríguez, Alicia Mohedano-Moriano, Begoña Polonio-López, Clara Maestre-Miquel, Antonio Viñuela, Carlos Durantez-Fernández, Miguel Á. Castro Villamor, José L. Martín-Conty

**Affiliations:** 1 Emergency Department, Hospital Universitario Rio Hortega, 47012 Valladolid, Spain; rlopeziz@saludcastillayleon.es; 2 Advanced Life Support Unit, Emergency Medical Services, 40002 Segovia, Spain; pdelbrioibaanezd@saludcastillayleon.es; 3 Advanced Life Support Unit, Emergency Medical Services, Advanced Clinical Simulation Centre, Faculty of Medicine, Universidad de Valladolid, 47005 Valladolid, Spain; 4 Faculty of Health Sciences, Universidad de Castilla la Mancha, 45600 Talavera de la Reina, Spain; Alicia.Mohedano@uclm.es (A.M.-M.); Begona.polonio@uclm.es (B.P.-L.); Clara.maestre@uclm.es (C.M.-M.); Antonio.vinuela@uclm.es (A.V.); Carlos.durantez@uclm.es (C.D.-F.); JoseLuis.MartinConty@uclm.es (J.L.M.-C.); 5 Advanced Clinical Simulation Centre, Faculty of Medicine, Universidad de Valladolid, 47005 Valladolid, Spain; mcastrovi@saludcastillayleon.es

**Keywords:** SOFA, qSOFA, clinical decision-making, early mortality, clinical deterioration, patient safety, emergency department

## Abstract

The objective of this study was to analyze and compare the usefulness of quick sequential organ failure assessment score (qSOFA) and sequential organ failure assessment (SOFA) scores for the detection of early (two-day) mortality in patients transported by emergency medical services (EMSs) to the emergency department (ED) (infectious and non-infectious). We performed a multicentric, prospective and blinded end-point study in adults transported with high priority by ambulance from the scene to the ED with the participation of five hospitals. For each score, the area under the curve (AUC) of the receiver operating characteristic (ROC) curve was calculated. We included 870 patients in the final cohort. The median age was 70 years (IQR 54–81 years), and 338 (38.8%) of the participants were women. Two-day mortality was 8.3% (73 cases), and 20.9% of cases were of an infectious pathology. For two-day mortality, the qSOFA presented an AUC of 0.812 (95% CI: 0.75–0.87; *p* < 0.001) globally with a sensitivity of 84.9 (95% CI: 75.0–91.4) and a specificity of 69.4 (95% CI: 66.1–72.5), and a SOFA of 0.909 (95% CI: 0.86–0.95; *p* < 0.001) with sensitivity of 87.7 (95% CI: 78.2–93.4) and specificity of 80.7 (95% CI: 77.4–83.3). The qSOFA score can serve as a simple initial assessment to detect high-risk patients, and the SOFA score can be used as an advanced tool to confirm organ dysfunction.

## 1. Introduction

Early warning scores (EWSs) have long been used for the prognostic assessment of patients in different healthcare settings. Several of them have been developed in intensive care units (ICUs) in order to assess the degree of organ dysfunction as accurately as possible and allow the prognoses of patients to be assessed throughout their hospitalization [1,2]. 

Among them, sequential organ failure assessment (SOFA) scores were initially developed to assess organ failure related to sepsis, with this being applied in recent years to a wide variety of clinical processes, especially in critical pathologies [3,4,5,6]. Following the new definition of sepsis in 2016 [7], the SOFA score is now used as the key criterion in the diagnosis of septic syndrome. Additionally, a new EWS called a quick sequential organ failure assessment score (qSOFA) was developed, which is a diagnostic tool used to quickly, easily and very reliably determine patients at high-risk of sepsis outside of ICUs [8,9]. In addition, SOFA scores are increasingly used to assess the response to therapies in the context of clinical trials [3,10].

Most studies have been carried out in ICUs [8,11,12], while very few have been carried out in emergency departments (EDs), and even fewer in non-infectious pathologies [13,14,15,16]. The use of these scores for non-infectious diseases outside ICUs has not yet been sufficiently studied, which can result in the underuse of these tools in hospital departments in many cases [10,17,18,19].

EDs provide healthcare to all types of patients in a wide range of clinical situations. Cases that are transferred to an ED by ambulance can be particularly complex. They are often highly complex and are very often pluripathological patients whose prognostic assessment is difficult.

In these special situations, systematic action by all healthcare workers involved in the care is essential. The use of EWSs could help with the initial assessment, which would result in safer decision-making [20,21,22].

Establishing organ dysfunction in patients is essential, and not only among patients with an infectious pathology. A delay in adequate advanced life support, as well as in ICU admission, has been associated with a higher probability of mortality [23]. In addition, high SOFA score values imply increased mortality, both in infectious and non-infectious patients [23]. Therefore, knowing that organ dysfunction is associated with mortality should require evaluation protocols in the ED and ICU, from the moment this dysfunction is assessed, something the SOFA score is able to establish in a simple way [24].

The primary objective of this study was to analyze and compare the usefulness of the qSOFA and SOFA scores for the detection of early (two-day) mortality, and secondarily to analyze this predictive capacity for medium-term (30-day) mortality in patients evacuated by emergency medical services (EMSs) and transferred to an ED with acute disease (infectious and non-infectious). In these contexts, we want to analyze whether the qSOFA and SOFA scores can be used as EWSs, with data collected in EDs during the first care of the sick patient.

## 2. Material and Methods

### 2.1. Study Design and Setting

A pragmatic, multicentric, prospective, controlled, and blinded end-point study of adults transported with high priority by ambulance to the ED between 1 October 2018 and 31 December 2019 was carried out.

This study was realized in the public health system of the Community of Castile and Leon (Spain), with the participation of five hospitals (Burgos University Hospital, Segovia Hospital Complex, Salamanca University Assistance Complex, Rio Hortega University Hospital and Valladolid University Clinic), four tertiary university hospitals and one small general district hospital, with a reference population of 1,351,962 inhabitants.

EDs operate 24/7, 365 days a year. Patients are received by an emergency registered nurse (ERN) in the triage area who performs the first evaluation, measuring the patient’s vital signs and assigning them a triage category. Then, depending on their priority, the patient is treated. A medical doctor (MD) performs the anamnesis and requests the necessary complementary tests. In emergency cases that require resuscitation, the patient goes directly to the critical area and receives standard advanced life support procedures.

This study was approved by the Research Ethics Committee (REC) of the public health system of Castile and Leon (REC number: #PI 18-010, #PI 18-895, #PI 18-10/119, #PI MBCA/dgc and #PI 2049). All patients (or guardians) signed an informed consent. This study is reported in line with the STROBE statement. The review protocol of this study was registered with ICTRP (doi.org/10.1186/ISRCTN17676798).

### 2.2. Population

The sample size was based on an expected 90% area under the receiver operating characteristic curve (AUROC) with a precision of 2% (with a 99% confidence level), requiring 788 subjects. A loss to follow-up rate of 5% was estimated, so the final estimated sample was 829 subjects.

Adult patients were recruited from among all cases of patients with acute disease transferred by EMS to the ED. Patients with a terminal status or with acute psychiatric pathology, pregnant women and cases of repeated attendance (only the first chronological event was counted) were excluded. Cases of cardiorespiratory arrest or death on arrival at the hospital were also excluded, as were cases in which a parameter for calculating the scores was missing.

During the initial contact at the scene or en route, an attempt was made to obtain informed consent. The ERN managed the primary consent process. If the patient presented an emergency situation or was not mentally capable of understanding the document, a further attempt was made to obtain consent in the ED. Finally, if none of the above were possible, a relative or legal guardian was contacted, and if informed consent could not be obtained after all of these attempts, the patient was excluded. The MD managed the secondary consent process.

### 2.3. Study Protocol and Data Abstraction

The main outcome variable was in-hospital early (two-day) mortality from any cause. The secondary outcome was 30-day in-hospital mortality.

A standardized case form (electronic medical record) was used. An ERN recorded the vital signs (respiratory rate, oxygen saturation, use of supplemental oxygen, blood pressure, heart rate, temperature and level of consciousness), and the ED subsequently recorded the analytical data, evolution of the patient, destination and diagnosis.

The parameters collected in the triage area were used to calculate the qSOFA score (see Table 1), while the SOFA score was calculated based on the results obtained during the first 8 h of the patient’s stay in the ED and the analytical parameters (platelets, bilirubin and creatinine) of the first blood sample collected. For mean arterial pressure or pulse oximetry saturation/fraction of the inspired oxygen ratio, we extracted the most abnormal value recorded within the first 8 h of their stay in the ED, not evaluating subsequent data (either obtained in the ED or during the subsequent hospitalization) (see Table 2).

For the calculation of the SOFA score, creatinine values (not urine production) and pulse oximetry saturation/fraction of the inspired oxygen ratio (not partial pressure of oxygen in the arterial blood/fraction of inspired oxygen) were used as a parameter to assess ventilatory function. Both creatinine and pulse oximetry saturation/fraction of the inspired oxygen ratio were routinely obtained in all patients, but not urine output or partial pressure of oxygen in the arterial blood/fraction of inspired oxygen.

By reviewing the electronic medical record 30 days after the index event (ED care), an associate researcher from each hospital obtained the hospital outcomes, including inpatients, ICU admission, two- and 30-day in-hospital mortality and final diagnosis catalogued as infectious or non-infectious pathology.

The entire research team received specific training on the objectives of the study, the standardized collection of data sets and how to calculate the scores. 

The following extractors were used to link the patients: day of admission, affiliation, gender, age and health card or identity document number. A specific database was created where patient data were recorded electronically. Once the database was purged through logic tests, detection of extreme values and searches for lost or unknown data took place. Before the statistical analysis, the database was anonymized.

### 2.4. Data Analysis

Quantitative variables are described as the median and interquartile range (25th–75th percentile). Normality tests were performed (Shapiro–Wilk and Anderson–Darling test). A Mann–Whitney U test was used to compare the locations of the quantitative variables. Qualitative variables are described as absolute frequencies with their percentages. To analyze the association between qualitative variables, a chi-square test for 2 × 2 and/or contingency tables for proportions were used. If necessary (percentage of boxes with expected values less than five but greater than 20%), we used Fisher’s exact test.

To determine the predictive capacity of the analyzed scores, the AUROC was calculated for the qSOFA and SOFA for two- and 30-day in-hospital mortality. For each score and outcome, the best cut-off (Youden’s index) was also calculated, with its corresponding sensitivity, specificity, positive predictive value, negative predictive value, positive likelihood ratio, negative likelihood ratio, odds ratio and diagnostic prediction. In all tests, a confidence level of 95% and a *p*-value of less than 0.05 were considered significant.

The data are presented according to the Standards for Reporting Diagnostic Accuracy 2015 statement [25]. Statistical analysis was performed with XLSTAT BioMED for Microsoft Excel version 14.4.0. (Microsoft Inc., Redmond, WA, USA).

## 3. Results

### 3.1. Patient Baseline

**Figure 1:**
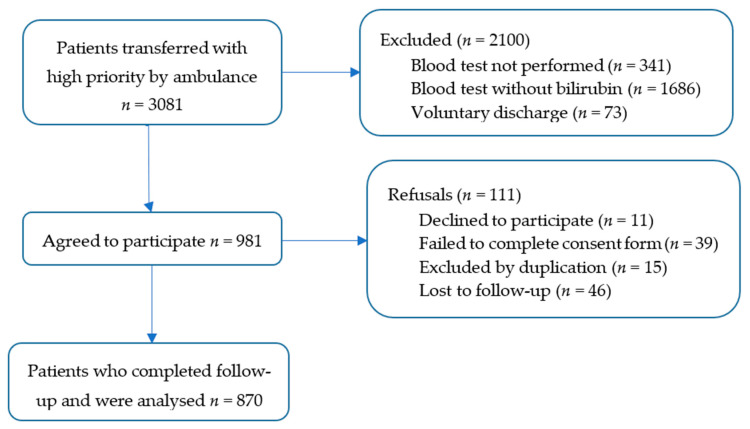
Flowchart of the study participants.

Between 1 October 2018 and 31 December 2019, a total of 3081 patients were transferred with high priority by ambulance to the EDs of the study referral hospitals. After applying the exclusion criteria and lost to follow-up, 870 patients were included in the final cohort (Figure 1).

The median age was 70 years (IQR 54–81 years), and 338 (38.8%) of the participants were women. Two-day mortality was 8.3% (73 cases), and 30-day mortality was 17.5% (152 cases), with 20.9% of cases having an infectious pathology. The characteristics of the patients are presented in Table 3.

### 3.2. Prognostic Accuracy of the Scores

**Figure 2:**
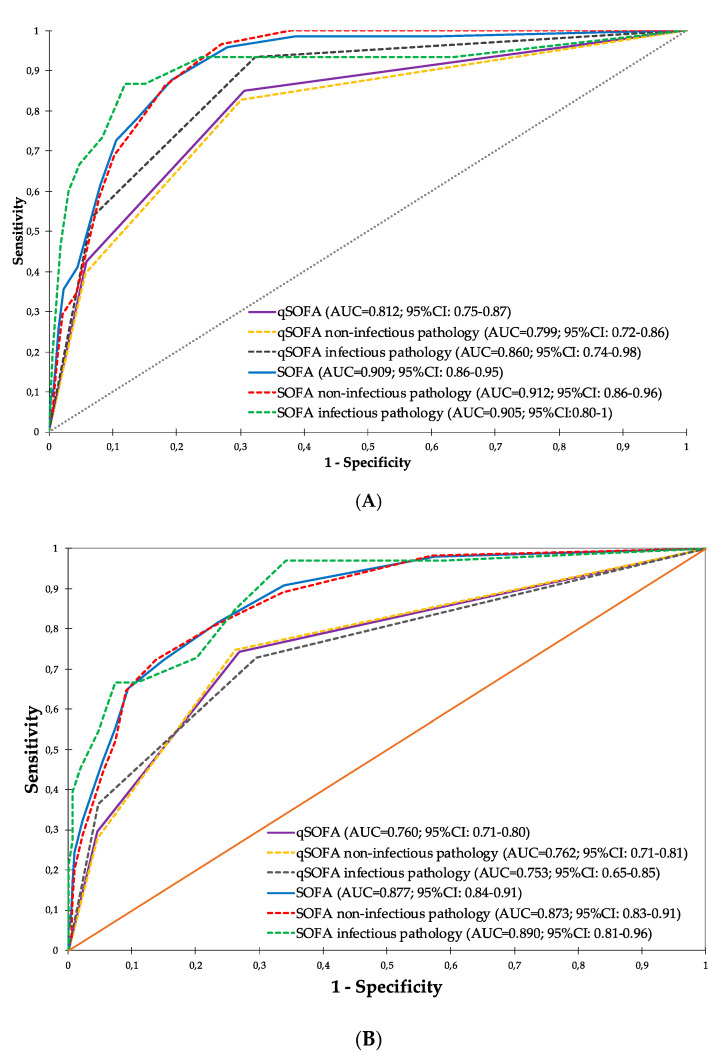
Diagnostic performance curves and areas under the curve with 95% confidence intervals for qSOFA and SOFA, globally, for non-infectious and infectious pathologies. (**A**) Two-day mortality; (**B**) 30-day mortality (in all cases *p* < 0.001). qSOFA: quick sequential organ failure assessment score; SOFA: sequential organ failure assessment score.

The prognostic accuracy of the scores for predicting two-day mortality is represented in Figure 2A. The qSOFA presented an area under the curve (AUC) of 0.812 (95% CI: 0.75–0.87) and a SOFA of 0.909 (95% CI: 0.86–0.95) globally. The qSOFA increased its predictive capacity in the infectious pathology cohort, with an AUC of 0.860 (95% CI: 0.74–0.98); however, the SOFA behaved excellently both in non-infectious and infectious pathologies. Regarding 30-day mortality, the qSOFA presented an AUC of 0.760 (95% CI: 0.71–0.80), and the SOFA, although its predictive performance decreased slightly, obtained an AUC of 0.877 (95% CI: 0.84–0.91), with similar values for both infectious and non-infectious pathologies (see Figure 2B).

### 3.3. Cut-Off Points for qSOFA and SOFA

For two-day mortality, the qSOFA presented an endpoint of two points, with a sensitivity of 84.9 (95% CI: 75.0–91.4) and specificity of 69.4 (95% CI: 66.1–72.5), while the SOFA (for a cut-off of four points) had a sensitivity of 87.7 (95% CI: 78.2–93.4) and a specificity of 80.7 (95% CI: 77.4–83.3). The high odds ratio of the SOFA score for non-infectious pathology stands out, as well as an excellent negative likelihood ratio. The results for the different outcomes for qSOFA and SOFA can be seen in Table 4. For all outcomes studied, a very high negative predictive value was maintained.

For 30-day mortality, the qSOFA presented a cut-off of two points, with a sensitivity of 74.3 (95% CI: 66.9–80.6) and specificity of 73.1 (95% CI: 69.8–76.2). In non-infectious and infectious pathologies, the qSOFA score obtained sensitivities of 74.6 (95% CI: 66.3–81.7) and 72.7 (95% CI: 55.8–84.9) and specificities of 73.8 (95% CI: 70.1–77.3) and 70.5 (62.7–77.2), respectively. The SOFA score presented a cut-off of three points for 30-day mortality, with a sensitivity of 81.6 (95% CI: 74.7–86.9) and a specificity of 76.5 (95% CI: 73.2–79.4). In non-infectious pathologies, the cut-off was four points, with a sensitivity of 72.3 (95% CI: 63.6–79.5) and a high specificity of 86.3 (83.2–88.9); however, in infectious pathologies there was an excellent sensitivity of 97.0 (84.7–99.5) and a discrete specificity of 65.8 (57.8–72.9), for a two-point cut-off.

## 4. Discussion

With this multicentric, prospective study in adults transported with high priority by ambulance to the ED, we observed that the SOFA score had an excellent ability to discriminate the patient’s risk both at two and 30 days, and was significantly better than that presented by the qSOFA score.

In recent years, interest in qSOFA and SOFA scores has increased significantly [9,26]. There have been a few studies that have analyzed the use of these scores in non-infectious pathologies [4,27], but, as far as we know, this is the first study to evaluate these scores in a cohort of patients transferred to the hospital by ambulance; that is to say, cases that have previously been evaluated and treated by EMS healthcare workers.

High scores in the ED for both scores have been significantly associated with high in-hospital mortality for all the intervals analyzed, which is in line with similar studies [27]. In the few studies in which in-hospital mortality was analyzed among the general population who attended an ED, the AUC was obtained in a range between 0.73 and 0.75, with values lower than the AUC found in our cohort [10,27]. Our cohort of patients was a sample that a priori were patients with severity criteria transferred by ambulance to the hospital, which means that our predictive capacity may have been slightly higher.

Other studies have observed a clear link between elevated SOFA score values performed in EDs and admission to the ICU [1,13,28]. Our study presented a lower number of ICU admissions compared to other studies, despite presenting high scores in the SOFA, which may be due to the characteristics of the sample [11,23]. Our population was mostly older adults (66% of the patients were older than 65 years), which is surely a restriction to admission to the ICU [29,30].

Undoubtedly, the qSOFA score is much simpler than the SOFA score, since with three simple parameters it is able to effectively discriminate the risk of early clinical deterioration, especially in patients with suspected infection. Establishing a two-point alert trigger in the qSOFA score as the cut-off point to determine a risk of mortality among all patients who arrive at an ED can help from the initial moments of care (triage) to identify high-risk patients, and not only among cases with suspected infection [7]. The qSOFA has a sensitivity greater than 0.80, both for infectious and non-infectious pathologies, which makes this simple score an excellent tool for a first approach to all types of patients.

As an alert trigger, the qSOFA is easy to apply, can be performed by personnel with minimal training, and is a score that does not require invasive tests. However, the SOFA score assesses organ dysfunction with greater precision, and provides us with a more accurate determination of the risk to the patient [23,31]. In addition, the SOFA score allows the continuous monitoring of the patient, so that their evolution and the effectiveness of the procedures performed can be known. A variation of a SOFA score (delta-SOFA) of two or more points is directly related to a worse prognosis, and not only in infectious pathologies and sepsis [7,24], but also among cases of non-infectious pathologies [23].

In the ED, professionals must make decisions quickly in critical situations. In these special circumstances, the use of scores can help to evaluate all patients homogeneously and not only those who evidently present warning signs and/or symptoms [32,33]. The qSOFA score represents the first assessment that is carried out quickly and non-invasively, while the SOFA score is a more sensitive tool that provides more information and is much more accurate, but requires complementary studies and an advanced level of training.

Our data indicate that a qSOFA score of two points or a SOFA score of four points in patients transferred with high priority by ambulance to the ED represents a high probability of risk of short-term clinical deterioration (within 48 h) both in cases of infectious and non-infectious pathologies.

### Limitations

Our study had several limitations. Firstly, the sample was chosen among patients transferred to the ED by EMSs during the study period. This assumes that they were patients who had already been previously evaluated by a medical team, and a certain severity is assumed for them to be transferred to the hospital. To minimize bias, patients were selected at all hours of the day, every day of the week, over more than one consecutive year.

Secondly, two- and 30-day in-hospital mortality were determined as the main outcomes, with us being aware that this time window was very limited and deaths occurring outside the hospital being excluded. Two-day mortality is directly related to the primary cause that motivated the care, and 30-day mortality is a very reliable indicator of hospital care in the medium term. Our outcomes were in line with similar publications [34,35].

Finally, larger prospective studies, with a sufficient number of patients, are required to be able to know the exact diagnostic accuracy of the qSOFA and SOFA scores, especially in non-infectious pathologies.

## 5. Conclusions

In summary, the qSOFA score can serve as a first-line evaluation to detect high-risk patients both in infectious and non-infectious patients, and the SOFA score can be used as an advanced tool to confirm organ dysfunction.

Patients evaluated and evacuated with high-priority by the EMS to an ED can benefit from the routine use of qSOFA during the first evaluation and SOFA during their complete evaluation

## Figures and Tables

**Table 1:** Quick sequential organ failure assessment score (qSOFA).

Criteria	Points
Respiratory rate ≥ 22/minute	1
Systolic blood pressure ≤ 100 mmHg	1
Change in mental status (GCS < 15 points)	1

GCS: Glasgow coma scale.

**Table 2:** Sequential organ failure assessment score (SOFA).

Variables	0	1	2	3	4
Respiratory	PaO_2_/FiO_2_ > 400 SpO_2_/FiO_2_ > 302	PaO_2_/FiO_2_ < 400 SpO_2_/FiO_2_ < 302	PaO_2_/FiO_2_ < 300 SpO_2_/FiO_2_ < 221	PaO_2_/FiO_2_ < 200 SpO_2_/FiO_2_ < 142	PaO_2_/FiO_2_ < 100 SpO_2_/FiO_2_ < 67
Cardiovascular (doses in mcg/kg/min)	MAP ≥ 70 mmHg	MAP < 70 mmHg	Dopamine ≤ 5 or any Dobutamine	Dopamine > 5 Norepinephrine ≤ 0.1	Dopamine > 15 Norepinephrine > 0.1
Liver, bilirubin (mg/dL)	<1.2	1.2–1.9	2–5.9	6–11.9	>12
Renal, creatinine (mg/dL)	<1.2	1.2–1.9	2.0–3.4	3.5–4.9	>5.0
Coagulation (platelets × 10^3^/mm^3^)	≥150	<150	<100	<50	<20
Neurologic (GCS, points)	15	13–14	10–12	6–9	<6

PaO_2_/FiO_2_ ratio: partial pressure of oxygen in arterial blood/fraction of inspired oxygen; SpO_2_/FiO_2_ ratio: pulse oximetry saturation/fraction of inspired oxygen ratio; MAP: mean arterial pressure; GCS: Glasgow coma scale.

**Table 3:** Demographic, clinical and hospital outcomes of included patients.

Characteristic ^1^	Total	2-Days Mortality	*p*-Value	30-Days Mortality	*p*-Value
Number [*n* (%)]	870 (100)	73 (8.3)		152 (17.5)	
Age (years) ^2^	70 (54–81)	73 (62–82)	0.007	73 (61–84)	<0.001
Female ^3^	338 (38.8)	26 (35.6)	0.554	57 (37.5)	0.707
Quick sequential organ failure assessment score					
Breathing rate (bpm) ^2^	15 (12–19)	17 (14–23)	0.055	15 (13–20)	0.362
SAP (mmHg) ^2^	125 (109–144)	116 (104–133)	0.007	121 (107–140)	0.107
GCS (points) ^2^	15 (14–15)	15 (13–15)	0.970	15 (14–15)	0.921
qSOFA (points) ^2^	1 (1–2)	2 (2–3)	<0.001	2 (1–3)	<0.001
Sequential organ failure assessment score					
SpO_2_/FiO_2_ ratio ^2^	443 (266–462)	443 (250–462)	0.771	443 (279–462)	0.843
MAP (mmHg) ^2^	89 (78–101)	82 (73–92)	<0.001	84 (76–98)	0.094
Inotropic agents ^3^	47 (5.4)	4 (5.5)	0.976	10 (6.6)	0.481
Bilirubin (mg/dL) ^2^	0.55 (0.38–0.85)	0.97 (0.80–1.12)	0.014	0.56 (0.43–0.82)	0.065
Creatinine (mg/dL) ^2^	0.99 (0.79–1.34)	0.97 (0.80–1.12)	0.046	0.99 (0.82–1.30)	0.084
Platelets (10^3^/mm^3^) ^2^	212 (166–269)	235 (183–285)	0.120	211 (177–264)	0.739
SOFA (points) ^2^	1 (1–4)	7 (5–10)	<0.001	6 (3–8)	<0.001
Hospital outcomes					
Inpatients ^3^	590 (67.7)	73 (100)	0.003	152 (100)	0.008
ICU admissions ^3^	214 (24.6)	32 (43.8)	<0.001	54 (35.5)	0.001
Infectious pathology ^3^	182 (20.9)	23 (31.5)	<0.001	47 (30.9)	<0.001
Non-infectious pathology ^3^	688 (79.0)	50 (68.5)	0.020	105 (69.1)	0.001

^1^ Values expressed as total number (fraction) and medians (25th–75th percentile) as appropriate; ^2^ The *p* values were calculated using a Mann–Whitney U-test (age, breathing rate, SAP, GCS, qSOFA, SpO_2_/FiO_2_ ratio, MAP, bilirubin, creatinine, platelets and SOFA); ^3^ The *p*-values were calculated using a Chi-square test (gender, inotropic agents, inpatients, intensive care unit (ICU) admission and pathology). SAP: systolic arterial pressure; GCS: Glasgow coma scale; qSOFA: quick sequential organ failure assessment score; SpO2: oxygen saturations; SpO_2_/FiO_2_ ratio: pulse oximetry saturation/fraction of the inspired oxygen ratio; MAP: mean arterial pressure; SOFA: sequential organ failure assessment score; ICU: intensive care unit.

**Table 4:** Cut-off points for combined sensitivity and specificity with the best score (Youden’s test) on the qSOFA and SOFA for two-day mortality.

Scores	Statistics	All Patients ^1^	Non-Infectious Pathology ^1^	Infectious Pathology ^1^
	Prevalence	0.084	0.084	0.082
qSOFA	Cut-off	2	2	2
	Se	84.9 (75.0–91.4)	82.8 (71.1–90.4)	93.3 (70.2–98.8)
	Sp%	69.4 (66.1–72.5)	69.8 (66.1–73.3)	67.7 (60.2–74.3)
	PPV	20.3 (16.1–25.1)	20.2 (15.6–25.7)	20.6 (12.7–31.6)
	NPV	98.0 (96.5–98.9)	97.8 (96.0–98.8)	99.1 (95.2–99.8)
	LR(+)	2.77 (2.41–3.20)	2.74 (2.32–3.24)	2.89 (2.23–3.74)
	LR(−)	0.22 (0.13–0.38)	0.25 (0.14–0.44)	0.10 (0.01–0.66)
	OR	12.77 (6.61–24.68)	11.12 (5.51–22.43)	29.30 (3.75–228.61)
	DA	70.7 (67.6–73.6)	70.9 (67.4–74.2)	69.8 (62.8–76.0)
SOFA	Cut-off	4	3	6
	Se	87.7 (78.2–93.4)	96.6 (88.3–99.0)	86.7 (62.1–96.3)
	Sp%	80.7 (77.4–83.3)	73.0 (69.4–76.3)	88.0 (82.2–92.1)
	PPV	29.4 (23.7–35.7)	24.8 (19.6–30.8)	39.4 (24.7–56.3)
	NPV	98.6 (97.4–99.3)	99.6 (98.4–99.9)	98.7 (95.2–99.6)
	LR(+)	4.54 (3.84–5.36)	3.58 (3.12–4.10)	7.24 (4.58–11.42)
	LR(−)	0.15 (0.08–0.28)	0.05 (0.01–0.18)	0.15 (0.04–0.55)
	OR	29.69 (14.46–60.97)	75.76 (18.29–313.88)	47.78 (10.04–227.42)
	DA	81.3 (78.5–83.7)	75.0 (71.6–78.1)	87.9 (82.4–91.9)

^1^ Bracketed numbers indicate 95% confidence interval. Se: sensitivity; Sp: specificity; PPV: positive predictive value: NPV: negative predictive value; LR: likelihood ratio; OR: odds ratio; DA: diagnostic accuracy; qSOFA: quick sequential organ failure assessment score; SOFA: sequential organ failure assessment score.
